# COVID-19 Impact on Alberta Nursing Home Workers: An Interpretive Descriptive Study With Direct Care Providers

**DOI:** 10.1093/geroni/igab046.1441

**Published:** 2021-12-17

**Authors:** Jude Spiers, Heather Titley, Amber Savage, Trina Thorne, Sandra Young, Neda Asadi, Corinne Schalm, Carole Estabrooks

**Affiliations:** 1 University of Alberta, Edmonton, Alberta, Canada; 2 University of Alberta, University of Alberta, Alberta, Canada; 3 University of Alberta, University of Alberta/ Edmonton, Alberta, Canada; 4 Ablerta Health, Alberta Health, EDMONTON, Alberta, Canada

## Abstract

COVID-19 has devastated the LTC sector, but we lack systematic information on the impact on frontline staff. Our research, a partnership with the continuing care branches of Alberta Health and Alberta Health Services, was aimed at assessing COVID-19 impacts on staffs’ well-being and quality of work-life and quality of care and life among residents. Here we report on staff. Using an interpretive descriptive approach, we interviewed 140 staff from January through April 2021, in 34 nursing homes. Facilities selected varied in ownership (public/private) and COVID-19 status (high, moderate, or low incidence). Virtual interviews focused on three key areas of impact: (a) staff mental and physical health, well-being, and work-life, (b) the facility, and (c) on residents. Interviews were analyzed using inductive content analysis. Dominant themes included a commitment of staff to resident wellbeing; a norm of stoicism in which accumulative stress of COVID-19 is recognized in participants’ private lives but not their work; the critical role of teamwork in managing extra workload associated with COVID-19 protocols; role flexibility, particularly managers’, enables workers to minimize interruptions to care activities; governmental wage subsidies and the restriction of workers to only one facility benefits residents and workers in terms of time and familiarity, but some health care aides faced a wage reduction of 30-40%. Alongside the research component, we regularly met with stakeholders and end-users to discuss emerging findings and potential areas needing urgent intervention, as well as longer-term programming as the impact of COVID-19 will persist for many years.

